# Linoleic Acid Induced Changes in SZ95 Sebocytes—Comparison with Palmitic Acid and Arachidonic Acid

**DOI:** 10.3390/nu15153315

**Published:** 2023-07-26

**Authors:** Dóra Kovács, Emanuela Camera, Szilárd Póliska, Alessia Cavallo, Miriam Maiellaro, Katalin Dull, Florian Gruber, Christos C. Zouboulis, Andrea Szegedi, Dániel Törőcsik

**Affiliations:** 1 Department of Dermatology, Faculty of Medicine, University of Debrecen, Nagyerdei krt. 98, 4032 Debrecen, Hungary; kovacs.dora@med.unideb.hu (D.K.); dull.kata@gmail.com (K.D.); aszegedi@med.unideb.hu (A.S.); 2 Laboratory of Cutaneous Physiopathology and Integrated Centre of Metabolomics Research, San Gallicano Dermatological Institute—IRCCS, 00144 Rome, Italy; emanuela.camera@ifo.it (E.C.); alessia.cavallo@ifo.it (A.C.); miriam.maiellaro@ifo.it (M.M.); 3 Genomic Medicine and Bioinformatic Core Facility, Department of Biochemistry and Molecular Biology, Faculty of Medicine, University of Debrecen, Nagyerdei krt. 98, 4032 Debrecen, Hungary; poliska@med.unideb.hu; 4 Research Division of Biology and Pathobiology of the Skin, Medical University of Vienna, Währinger Gürtel 18-20, 1090 Vienna, Austria; florian.gruber@meduniwien.ac.at; 5 Departments of Dermatology, Venereology, Allergology and Immunology, Staedtisches Klinikum Dessau, Brandenburg Medical School Theodor Fontane and Faculty of Health Sciences Brandenburg, Auenweg 38, 06847 Dessau, Germany; christos.zouboulis@mhb-fontane.de; 6 ELKH-DE Allergology Research Group, Nagyerdei krt. 98, 4032 Debrecen, Hungary

**Keywords:** linoleic acid, arachidonic acid, palmitic acid, sebocytes, gene expression changes, lipid profile

## Abstract

Linoleic acid (LA) is an essential omega-6 polyunsaturated fatty acid (PUFA) derived from the diet. Sebocytes, whose primary role is to moisturise the skin, process free fatty acids (FFAs) to produce the lipid-rich sebum. Importantly, like other sebum components such as palmitic acid (PA), LA and its derivative arachidonic acid (AA) are known to modulate sebocyte functions. Given the different roles of PA, LA and AA in skin biology, the aim of this study was to assess the specificity of sebocytes for LA and to dissect the different roles of LA and AA in regulating sebocyte functions. Using RNA sequencing, we confirmed that gene expression changes in LA-treated sebocytes were largely distinct from those induced by PA. LA, but not AA, regulated the expression of genes related to cholesterol biosynthesis, androgen and nuclear receptor signalling, keratinisation, lipid homeostasis and differentiation. In contrast, a set of mostly down-regulated genes involved in lipid metabolism and immune functions overlapped in LA- and AA-treated sebocytes. Lipidomic analyses revealed that the changes in the lipid profile of LA-treated sebocytes were more pronounced than those of AA-treated sebocytes, suggesting that LA may serve not only as a precursor of AA but also as a potent regulator of sebaceous lipogenesis, which may not only influence the gene expression profile but also have further specific biological relevance. In conclusion, we have shown that sebocytes are able to respond selectively to different lipid stimuli and that LA-induced effects can be both AA-dependent and independent. Our findings allow for the consideration of LA application in the therapy of sebaceous gland-associated inflammatory skin diseases such as acne, where lipid modulation and selective targeting of AA metabolism are potential treatment options.

## 1. Introduction

The sebaceous gland (SG), together with the hair follicle, forms the pilosebaceous unit whose primary function is to produce and secrete the lipid-rich sebum that lubricates the hair and moisturises the skin. However, sebum can also regulate several biological processes such as antimicrobial activity or photoprotection [1]. For its ubiquitous production, sebocytes take up lipids from the circulation and metabolise them, resulting in a unique and specific lipid profile of human sebum. This is composed of triglycerides (TGs); free fatty acids (FFAs), including arachidonic (AA), linoleic (LA), palmitic (PA), stearic and oleic acids; wax esters; squalene; and cholesterol [2,3,4,5,6,7,8]. We have previously reported that sebum lipids may not only coat the skin but also penetrate into the dermis to interact with various skin cell types such as macrophages and, presumably, sebocytes [5]. 

Among the sebum components, LA is central. It contributes to the structural organisation and function of the permeability barrier of the stratum corneum [9] and also stimulates lipid production, increasing cell size and promoting differentiation and the delayed proliferation of sebocytes [10,11,12]. In pathological conditions such as acne—one of the most common inflammatory skin diseases associated with SGs—reduced levels of LA compared with other species have been associated with increased microcomedone formation and increased follicular cast size in affected patients. Consistent with this, topically applied LA, in addition to the anti-inflammatory effects observed in *Cutibacterium acnes* (*C. acnes*)-activated macrophages [5,13,14,15], had a beneficial comedolytic effect in acne-prone patients by reducing microcomedones [15]. In contrast, the sebum of acne patients contains elevated levels of PA [16], which may also play a role in the pathogenesis of acne by enhancing the secretion of interleukin (IL)-1β in in vitro differentiated monocyte-derived macrophages [5] and the production of IL-6 and IL-8 by human sebocytes [6,17].

Through the expression of fatty acid desaturase 2 (FADS2), sebocytes are able to convert LA to AA [18]. As another prominent polyunsaturated fatty acid (PUFA) in the skin, AA is involved in the formation of neutral lipids and phospholipids and is an important regulator of sebocyte differentiation and lipid metabolism [19]. Meanwhile, as a precursor of leukotrienes and prostaglandins, AA is also involved in inflammatory responses [20]. Importantly, the enzymes that metabolise AA are also active in the SG and their expression is increased in acne-affected skin [21]. In addition, AA and its metabolites may also be important in the wound healing process, re-epithelialisation and angiogenesis, as suggested by mouse model studies [22].

Although a potential role for the LA–AA–AA derived keto-metabolite axis (e.g., 5-oxo-6E,8Z,11Z,14Z-eicosatetraenoic acid [5-KETE], 12-oxo-5Z,8Z,10E,14Z-eicosatetraenoic acid [12-KETE]) in the regulation of SG differentiation and lipid synthesis was strongly supported in a previous publication [19], there were also differences in the effects of each lipid. When assessing the levels of inflammatory cytokines measured, LA strongly up-regulated IL-6 secretion, whereas AA induced only a mild release of IL-6 and IL-8 from SZ95 sebocytes [18]. Regarding the expression of mRNA and protein levels of key lipid metabolising enzymes, such as FADS2 and stearoyl-CoA desaturase (SCD), which catalyses the rate-limiting step in the formation of monounsaturated fatty acids, e.g., the formation of oleic acid from the saturated fatty acid stearic acid, LA induced the expression of both enzymes. In contrast, AA increased SCD mRNA levels but decreased FADS2 mRNA levels [18]. Further in vivo studies, which found increased levels of AA and its derivatives but decreased levels of LA in skin samples from acne patients [19], have suggested that the different players in the proposed axis may indeed have different biological properties in both SG biology and acne development [23]. 

The aim of this study was therefore to assess the specific response of sebocytes to LA and to dissect the different roles of LA, PA and AA in regulating sebocyte functions at the level of gene expression and lipid production.

## 2. Materials and Methods

### 2.1. Cell Culture and Treatments 

Immortalized human SZ95 sebocytes [24] were cultured in Sebomed^®^ Basal Medium (Biochrom, Cambridge, UK), supplemented with 10% fetal bovine serum (FBS; Biosera, Nuaille, France), 1 mM CaCl_2_, 5 ng/mL human epidermal growth factor (EGF; Sigma-Aldrich, St. Louis, MO, USA), 500 U/mL penicillin and 0.5 mg/mL streptomycin (Sigma-Aldrich). Sebocytes were treated with 150 µM PA, 1 µM LA and 1 µM AA (Sigma-Aldrich), dissolved in ethanol:dimethyl sulfoxide (DMSO) in 1:1 ratio for 24 h. Control cells were treated with ethanol:DMSO in 1:1 ratio for 24 h (vehicle control). Cells were harvested after treatments and samples were stored at −20 °C until further analysis. 

### 2.2. Determination of mRNA Levels 

SZ95 sebocytes were cultured in 6-well plates in the presence of 150 µM PA, 1 µM LA or 1 µM AA for 24 h and total RNA was isolated using TRI Reagent (MRC, Cincinnati, OH, USA) according to the manufacturer’s protocol and quantified by using NanoDrop 2000 (Thermo Fisher Scientific, Walthman, MA, USA).

For RNA sequencing (RNA-Seq) and library preparation cDNA was generated from 1 µg total RNA using TruSeq RNA Sample Preparation Kit (Illumina, San Diego, CA, USA) according to the manufacturer’s protocol. Briefly, poly-A tailed RNAs were purified using oligodT-conjugated magnetic beads and fragmented at 94 °C degree for 8 min, then first strand cDNA was transcribed using random primers and SuperScript II reverse transcriptase (Life Technologies, Carlsbad, CA, USA). Following this step second-strand cDNA was synthesized, double-stranded cDNA end was repaired and 3′ ends were adenylated. Illumina index adapters were ligated. After adapter ligation enrichment PCR was performed to amplify adapter ligated cDNA fragments. Fragment size distribution and molarity of libraries were checked on an Agilent BioAnalyzer DNA1000 chip (Agilent Technologies, Santa Clara, CA, USA). The concentration of RNA-Seq libraries was diluted to 10 nM and 5 libraries were pooled prior to sequencing. Single read 50 bp sequencing was performed on the Illumina HiScan SQ instrument (Illumina). Each library pool was sequenced in one lane of the sequencing flow cell, yielding 16–18 million reads per sample. 

### 2.3. RNA-Seq Data Analysis 

Raw sequencing data (fastq) were aligned to the human reference genome version GRCh38 using the HISAT2 algorithm and BAM files were generated. Downstream analysis was performed using StrandNGS software (version 2.8, build 230243; Strand Life Sciences, Bangalore, India). BAM files were imported into the software, the DESeq algorithm was used for normalization and normalized expression data were used for statistical analysis. Biological process and reactome pathway analyses were performed using the Cytoscape 3.9.0 software with the ClueGO v2.5.8 plug-in [25]. Genes were normalized to vehicle control and results are expressed as average mean of triplicate samples. A heat map showing the replicates separately is provided in Figure S1. Gene expression data of differentially up- and down-regulated genes were filtered with 2-fold change. RNA-Seq data are available in the Sequence Read Archive (SRA) database under the following accession numbers: PRJNA646337 for PA and PRJNA882620 for LA and AA. Further functional clustering of the differentially expressed genes was performed using the Ingenuity Pathway Analysis software (Qiagen, Hilden, Germany, IPA Spring Release (2023) https://digitalinsights.qiagen.com/products/qiagen-ipa/latest-improvements/current-line/).

### 2.4. Lipid Analyses 

Lipids were extracted from SZ95 sebocytes as previously described [26]. Cell pellets were suspended in distilled water and cracked by triple freeze and thaw cycles. After centrifugation, the protein content of the supernatant was determined by Bradford’s assay. The membrane pellet was thoroughly mixed with the supernatant and added with butylhydroxytoluene 0.001% (Sigma-Aldrich) in methanol (MeOH) to prevent oxidation of oxygen sensitive compounds. To control the analytical processes and calculate lipid amounts, samples were spiked with a mixture of deuterated (d) internal standards (iSTD), including d6-cholesterol, d6-squalene, d17-C16:0, d31-Cer[NS]34:1, d98-TG48:0 and d7-phospholipids from the EquiSPLASH-LIPIDOMIX (Avanti Polar Lipids, Alabaster, AL, USA), i.e., d7-PC 33:1, and d7-PE 33:1, and d9-sphingomyelin, all binding deuterated oleic acid (d9–18:1). For liquid/liquid extraction, 1 mL ethyl acetate was added to the sample and vortexed for 2 min. After a centrifugation step to separate the phases, the upper organic solution enriched in cell lipids was collected in Eppendorf tubes and dissolved in 200 µL isopropyl alcohol (iPrOH). For gas chromatography–mass spectrometry (GCMS) analysis, 20 µL of the lipid extract from SZ95 sebocytes was transferred into a glass insert and dried under nitrogen. The dry extract was derivatized with 20 µL of N,O-bis(trimethyl-silyl)-trifluoroacetamide (BSTFA) added to 1% trimethylchlorosilane (TCMS) in pyridine. The reaction was carried out at 60 °C for 60 min to obtain TMS derivatives. Lipid extracts were mixed with MeOH at a dilution factor of 1:4 for LCMS analysis. The GCMS analysis was performed for the quantitative determination of FFAs, fatty alcohols (FAOHs), cholesterol and vitamin E. The instrument used was an 8890 GC System connected to a 5977B Series MSD single quadrupole (Agilent Technologies). The separation was performed on the HP-5MS UI fused silica column chemically bonded with a 5%-phenyl-methylpolysiloxane phase, 30 m × 0.250 mm (i.d.) × 0.25 µm film thickness (Agilent Technologies). The carrier gas (helium) flow rate was 1.2 mL/min. The total run time was 49 min. The derivatized lipid extracts were started to elute at an oven temperature of 80 °C, reaching 280 °C in 33 min and 310 °C in 12 min [27]. GCMS data were acquired using MassHunter GC/MSD 5977B acquisition software (version 3.1.199) in scan mode following EI ionization. The operating conditions were: 230 °C MS Source and 150 °C MS Quad, 70 eV electron impact voltage, 50–600 mass range and 3.125 scan speed (units/s). 

High-performance liquid chromatography (HPLC) analysis was performed on the Infinity II 1260 series HPLC system (Agilent Technologies) equipped with a degasser, a quaternary pump, an autosampler and a column compartment. 

Reversed phase HPLC (RP-HPLC) was the separation technique used to analyse TGs, diglycerides (DGs), cholesteryl esters (CEs) and ceramides under positive electrospray ionization ((+)ESI) conditions. A Zorbax SB-C8 column (2.1 mm × 50 mm, 1.8 µm particle size) (Agilent Technologies), thermostatted at 60 °C, with a maximum operating pressure of 600 bar/9000 psi, was used for the RP-HPLC separation. Cell extracts were eluted with a quaternary gradient of (A) 5 mM ammonium formate in MilliQ water (18.2 Ω), (B) MeOH, (C) acetonitrile (ACN), (D) iPrOH. The elution programme was 60% A, 28% B, 8% C, 4% D 0–10 min; 1% A, 70% B, 20% C, 9% D 10–20 min; 60% A, 28% B, 8% C, 4% D 20–22 min. A 2 min post-run of the initial condition was added. The flow rate was 0.4 mL/min and the injection volume was 0.8 µL. 

Hydrophilic interaction liquid chromatography (HILIC) in (+)ESI mode was used for the separation and quantification of polar lipids, mainly represented by phosphatidylcholines (PCs), phosphatidylethanolamines (PEs) and sphingomyelins (SMs). The HILIC separation was performed on a HALO HILIC column (Advanced Materials Technology, Phoenix, AZ, USA), 2.1 mm × 50 mm, 2.7 µm particle size, with a maximum operating pressure of 600 bar/9000 psi. The column temperature was adjusted to 40 °C. The mobile phase consisted of (A) 5 mM ammonium formate in MilliQ water (18.2 Ω), (B) MeOH and (C) ACN; the flow rate was 0.4 mL/min. The elution programme was 1% A, 2% B, 97% C, 0–18 min; 18% A, 2% B, 80% C, 18–20 min; 1% A, 2% B, 97% C, 20–22 min. A 2 min post run of the initial condition was added, the injection volume was 0.4 µL. 

A 6545 Quadrupole Time of Flight mass spectrometer (Q-TOF) (Agilent Technologies) was used as the high-resolution mass analyser. The instrument was connected to the HPLC system via an ESI Dual Agilent Jet Stream (AJS) interface with nitrogen used as nebulizing, sheath, drying, and collision gas. The temperature of the ion source gas was set at 200 °C. The flow rate and pressure of the nebulizer were set at 10 L/min and 30 psi, respectively. The sheath gas temperature and flow rate were set to 350 °C and 12 L/min, respectively. The capillary voltage parameter was 4000, the nozzle voltage parameter was 0. The fragmentor voltage parameter was 120 V and the skimmer voltage parameter was 40 V. Lipidomics data were collected in All Ions MS/MS mode at multiple collision energies (0, 20, 40 V). The *m*/*z* range for MS and MS/MS was 59–1700 at a mass resolution of 40,000. The instrument was calibrated at least daily prior to each series of samples analysed. Reference standards (Agilent Technologies) of mass 121.050873 and 922.009798 were used as internal mass calibrations to correct for low and high masses, respectively. LC-HRMS data were acquired and deconvoluted using the MassHunter Data Acquisition Software (version B.09.00 Build 9.0.9044.0, Agilent Technologies).

GCMS were processed using MassHunter Workstation Software Quantitative Analysis (version 10.1, Agilent Technologies), whereas LCMS data were processed using MassHunter Workstation Profinder (version 10.0, Agilent Technologies). 

### 2.5. Statistical Analyses 

Enrichment/depletion (two-tailed hypergeometric test) and Bonferroni step-down were used to analyse RNA-seq data using the Cytoscape 3.9.0 software with the ClueGO v2.5.8 plug-in. Terms with *p* ≤ 0.05 after the Bonferroni step-down correction for multiple testing are shown. All lipidomics data were derived by normalizing the response of the individual lipid by the response of the same-class labelled iSTD and multiplied by the pmole of the iSTD. Quantities of selected analytes were normalized by the protein content. Targeted data exploration and statistical analysis were performed at pmol/mg protein using Mass Profiler Professional software (version 15.1, Agilent Technologies). The abundance profiles of vehicle controls and SZ95 sebocytes treated with LA and AA were evaluated using volcano plots and hierarchical clustering analysis. An unpaired *t*-test was used to examine significant differences between vehicle controls and SZ95 sebocytes treated with LA or AA (*p* ≤ 0.05, FC ≥ 2.0). Statistical analysis of selected saturated and unsaturated fatty acids and cholesterol levels determined by GCMS was performed by one-way ANOVA followed by Tukey post hoc and Kruskal–Wallis tests followed by Dunn’s post hoc test. Data are expressed as mean ± SD. Statistical calculations were performed using GraphPad Prism 7 (version 7.0.0.159, GraphPad Software Inc., San Diego, CA, USA).

## 3. Results

### 3.1. Linoleic Acid Regulates a Specific Set of Genes in SZ95 Sebocytes

**Figure 1:**
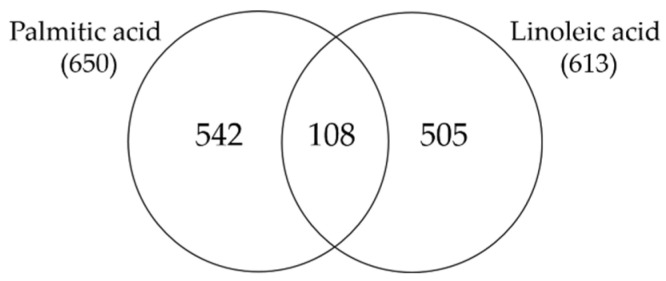
Venn diagram showing the number of genes which are significantly up- and down-regulated in SZ95 sebocytes after 24 h of PA and LA treatment as determined by our RNA-seq analysis.

As a starting point for our studies, we wanted to assess whether LA is a specific stimulus or rather a general lipid signal for sebocytes. Therefore, we identified genome-wide transcriptional changes by RNA-seq analysis in SZ95 sebocytes after 24 h of LA treatment, and compared them with the genetic program influenced by PA treatment from our previous experiments which was also performed on SZ95 sebocytes treated for 24 h [6]. Using Venn diagrams to visualize the genes differentially up- and down-regulated in response to LA and PA, our analyses revealed that while 505 genes were selectively induced by LA, only 108 genes were commonly regulated by both stimuli (Figure 1). 

**Figure 2:**
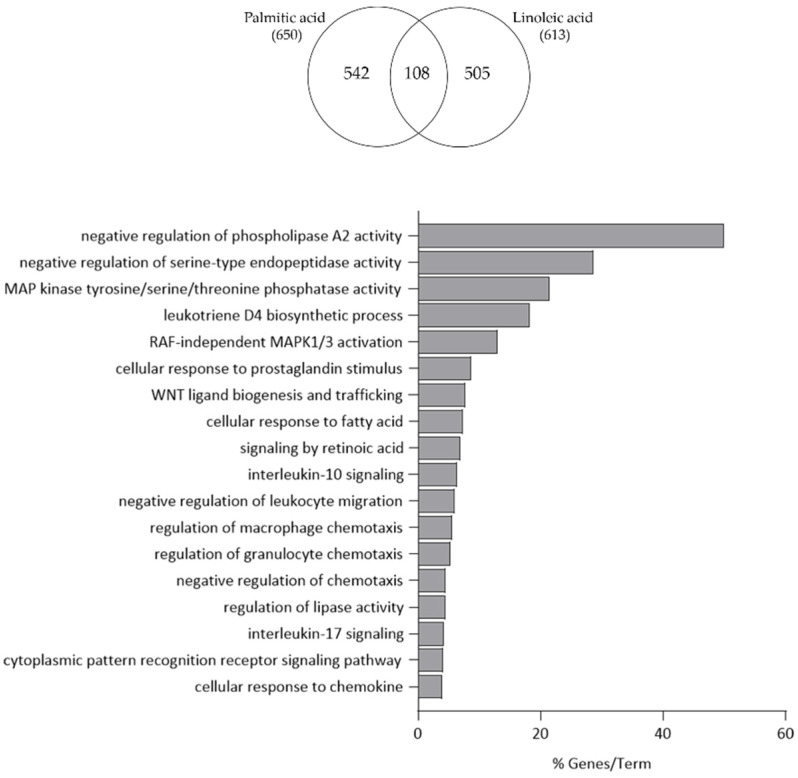
Functional clustering of the 108 transcripts up- and down-regulated in both PA- and LA-treated SZ95 sebocytes after 24 h. The y-axis represents the statistically significant terms of the biological process and reactome pathway analyses while the x-axis shows the percentage of genes belonging to a given term. Term *p*-value ≤ 0.05.

Functional clustering of the 108 commonly regulated transcripts by PA and LA treatment recognized phospholipase A2 and serine-type endopeptidase activity, mitogen-activated protein kinase (MAPK) signalling, leukotriene D4 biosynthetic process, cellular response to prostaglandin stimulus, WNT ligand biogenesis, retinoic acid signalling, leukocyte migration and macrophage/granulocyte chemotaxis, and IL-10 and -17 signalling (Figure 2). 

### 3.2. Linoleic Acid-Induced Gene Expression Changes in SZ95 Sebocytes 

To gain a deeper insight into the overall gene expression changes caused by LA in SZ95 sebocytes, further biological process and pathway analyses were performed on the 613 up- and down-regulated transcripts. 

**Figure 3:**
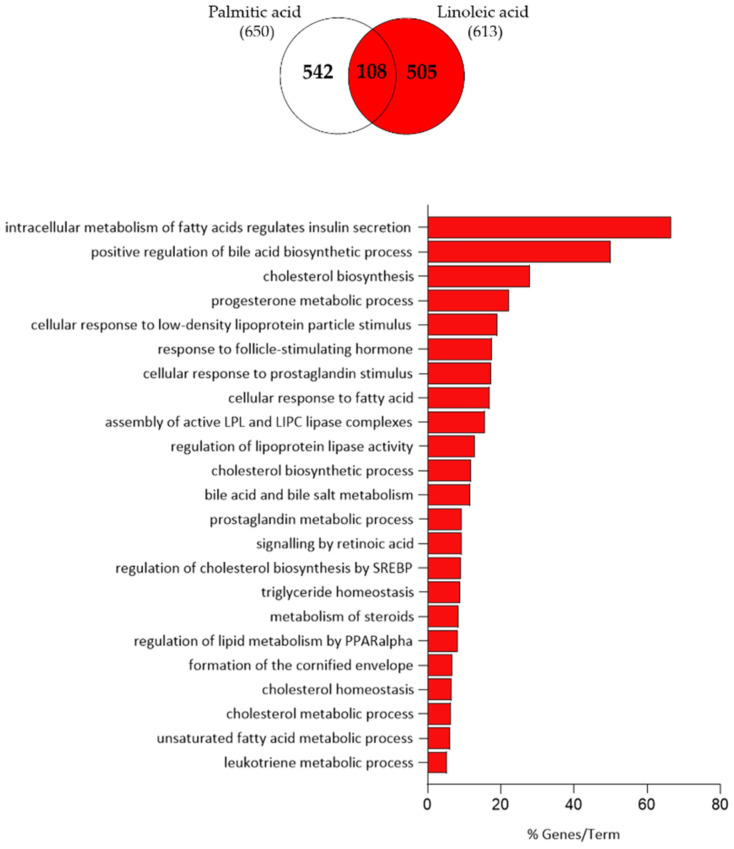
Functional clustering of the 212 transcripts up-regulated in LA-treated SZ95 sebocytes after 24 h (highlighted in red in the Venn diagram). The y-axis represents the statistically significant terms of the biological process and reactome pathway analyses while the x-axis shows the percentage of genes belonging to a given term. Term *p*-value ≤ 0.05.

LA treatment resulted in 212 up-regulated transcripts involved in the intracellular metabolism of fatty acid, bile acid and cholesterol biosynthesis, progesterone metabolism, cellular response to low-density lipoprotein particle/follicle-stimulating hormone/prostaglandin, lipoprotein lipase activity, retinoic acid signalling, regulation of cholesterol biosynthesis by sterol regulatory element-binding protein (SREBP), steroid metabolism, regulation of lipid metabolism by peroxisome proliferator-activated receptor (PPAR)-α, cornified envelope formation and leukotriene metabolism (Figure 3). 

**Figure 4:**
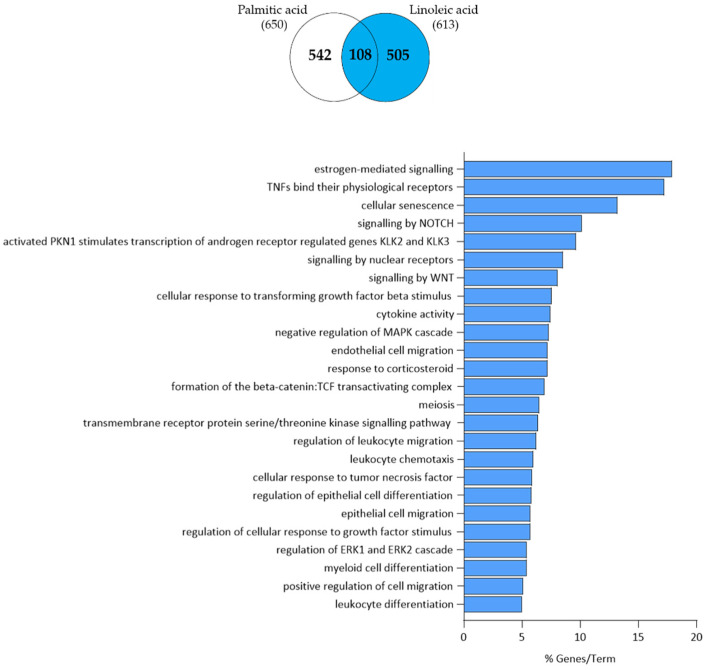
Functional clustering of the 401 down-regulated transcripts (highlighted in blue in the Venn diagram) in LA-treated SZ95 sebocytes after 24 h. The y-axis represents the statistically significant terms of the biological process and reactome pathway analyses while the x-axis shows the percentage of genes belonging to a given term. Term *p*-value ≤ 0.05.

The 401 down-regulated transcripts could be grouped into oestrogen-mediated signalling, tumour necrosis factors (TNFs) signalling, androgen and nuclear receptor signalling, WNT signalling, cellular response to transforming growth factor-β (TGF-β) stimulus, cytokine activity, regulation of the MAPK cascade, endothelial cell migration, response to corticosteroid, beta-catenin formation, regulation of leukocyte and epithelial cell migration/differentiation and chemotaxis, and regulation of the extracellular signal-regulated kinases (ERK) 1/2 cascade (Figure 4). 

These results suggest that the genes up-regulated by LA are mainly involved in lipid and steroid metabolism, while the down-regulated genes are associated with immune processes and differentiation in SZ95 sebocytes. 

### 3.3. Comparison of Linoleic Acid and Arachidonic Acid-Induced Gene Expression Changes in SZ95 Sebocytes 

**Figure 5:**
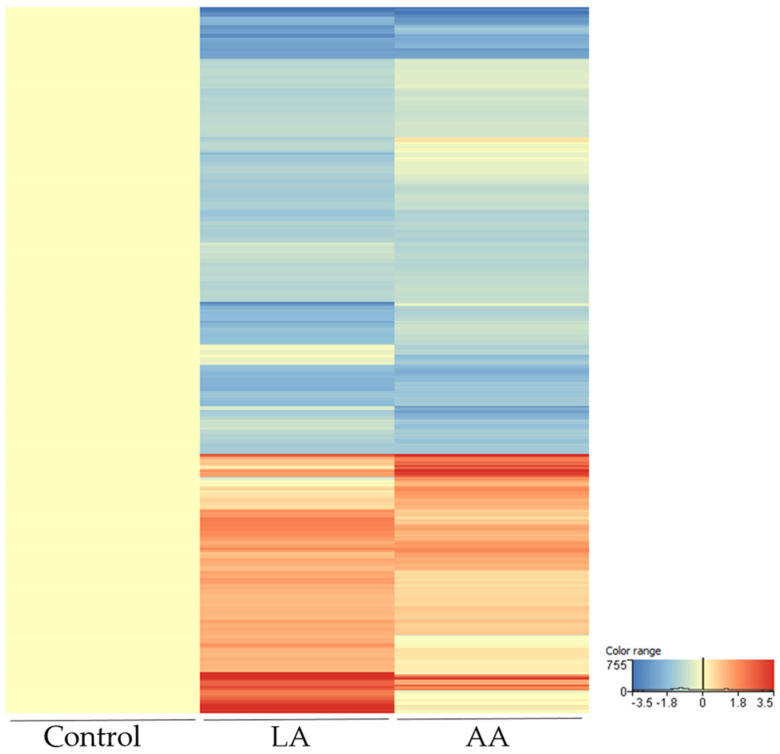
Differentially expressed genes shown as a heatmap in LA- and AA-treated SZ95 sebocytes for 24 h as determined by our RNA-seq analysis. Genes were normalized to vehicle control and results are expressed as the average of triplicate samples. Colour intensities reflect the ratio of signal intensities as shown.

Since LA is converted to AA [28,29], the next step was to focus on the LA–AA axis. The generated heat map shows the differentially up- and down-regulated transcripts at 24 h after LA and AA treatment, filtered by 2-fold change as observed in our RNA-seq analysis (Figure 5). 

**Figure 6:**
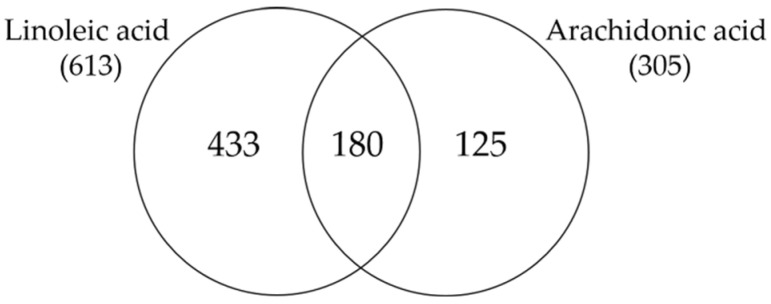
Venn diagram showing the number of genes which are significantly up- and down-regulated in SZ95 sebocytes after 24 h of LA and AA treatment as determined by RNA-seq analysis.

We found that of the 613 genes that were regulated by LA, 180 were also regulated by AA (Figure 6), suggesting that these are the genes that may be regulated via the LA–AA axis.

**Figure 7:**
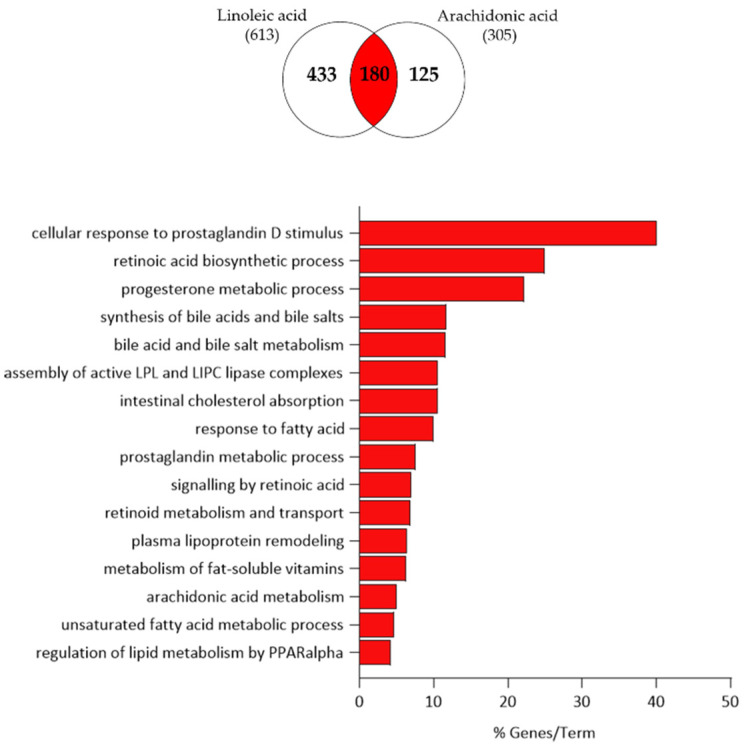
Functional clustering of the 29 up-regulated transcripts (highlighted in red in the Venn diagram) in both LA- and AA-treated SZ95 sebocytes after 24 h. The y-axis represents the statistically significant terms of the biological process and reactome pathway analyses while the x-axis shows the percentage of genes belonging to a given term. Term *p*-value ≤ 0.05.

To gain a deeper insight into the gene expression changes induced by LA and AA in SZ95 sebocytes, further pathway analysis of the commonly up- and down-regulated transcripts was performed. Among the 180 commonly regulated transcripts, we found that 29 transcripts involved in cellular response to prostaglandin D stimulus, retinoic acid biosynthesis, progesterone metabolism, synthesis of bile acid and bile salt, intestinal cholesterol absorption, response to fatty acid, plasma lipoprotein remodelling, arachidonic acid metabolism and regulation of lipid metabolism by PPAR-α were up-regulated (Figure 7).

**Figure 8:**
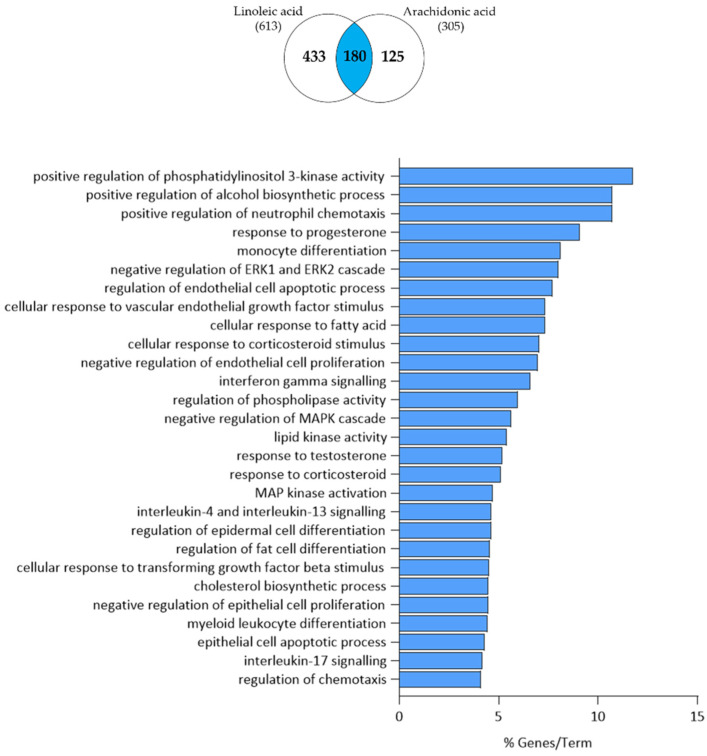
Functional clustering of the 150 down-regulated transcripts (highlighted in blue in the Venn diagram) in both LA- and AA-treated SZ95 sebocytes after 24 h. The y-axis represents the statistically significant terms of the biological process and reactome pathway analyses while the x-axis shows the percentage of genes belonging to a given term. Term *p*-value ≤ 0.05.

Clustering of the 150 commonly down-regulated genes revealed that such an axis may be responsible for the regulation of phosphatidylinositol 3-kinase (PI3K) activity, alcohol biosynthetic process, regulation of neutrophil chemotaxis, response to progesterone, monocyte differentiation, regulation of ERK1/2 cascade, cellular response to TGF-β, vascular endothelial growth factor (VEGF) stimulus and fatty acid, interferon-γ (IFN-γ) signalling, regulation of the MAPK cascade, response to testosterone and corticosteroid, IL-4/IL-13 signalling, regulation of fat cell differentiation, cholesterol biosynthetic process, regulation of epithelial cell proliferation and myeloid leukocyte differentiation, and IL-17 signalling (Figure 8). 

### 3.4. Arachidonic Acid-Independent Effects of Linoleic Acid 

The genes that were only regulated by LA, but not by AA, suggest that LA alters the expression of these genes independently of AA. Of the 433 genes regulated by LA but not by AA, 183 were up-regulated and 251 were down-regulated. 

**Figure 9:**
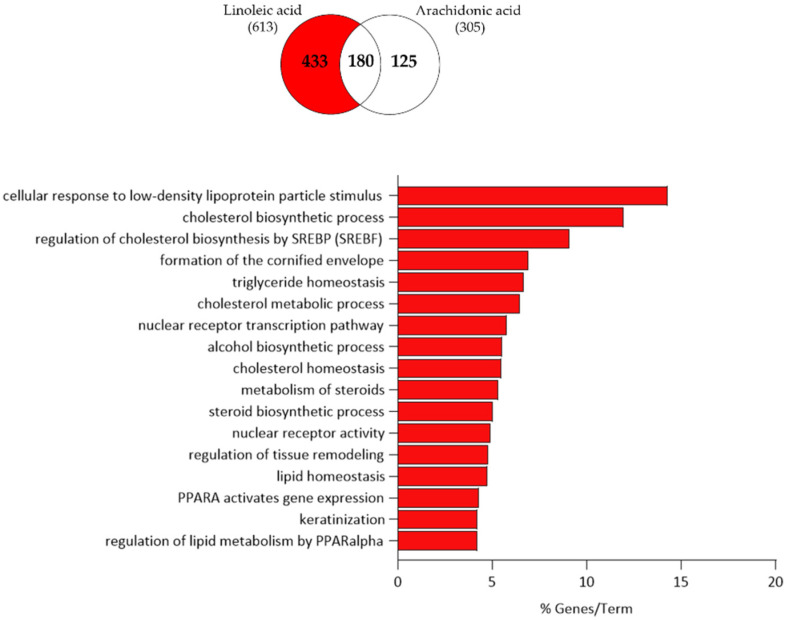
Functional clustering of the 183 up-regulated transcripts (highlighted in red in the Venn diagram) that were only regulated by LA in SZ95 sebocytes after 24 h. The y-axis represents the statistically significant terms of the biological process and reactome pathway analyses while the x-axis shows the percentage of genes belonging to a given term. Term *p*-value ≤ 0.05.

Functional clustering of the 183 up-regulated transcripts suggested that LA may modulate sebocyte functions such as cholesterol biosynthesis by SREBP, formation of the cornified envelope, triglyceride homeostasis, nuclear receptor transcription pathway, alcohol biosynthetic process, steroid metabolism, regulation of tissue remodelling, lipid homeostasis and metabolism by PPAR-α and keratinization (Figure 9).

**Figure 10:**
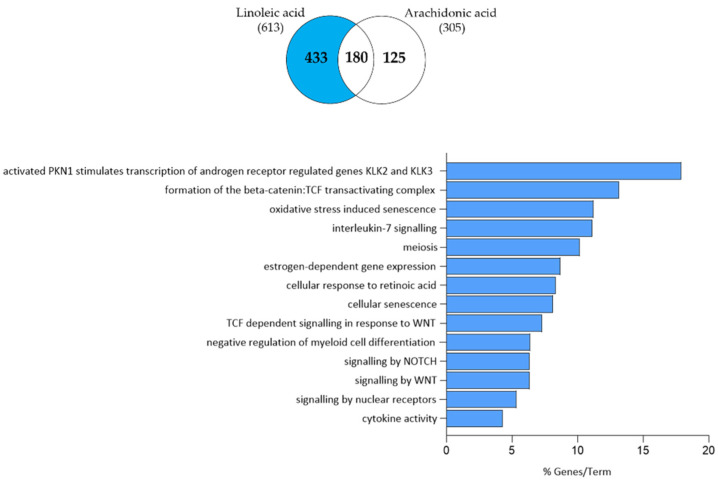
Functional clustering of the 251 down-regulated transcripts (highlighted in blue in the Venn diagram) that were only regulated by LA in SZ95 sebocytes after 24 h. The y-axis represents the statistically significant terms of the biological process and reactome pathway analyses, while the x-axis shows the percentage of genes belonging to a given term. Term *p*-value ≤ 0.05.

The 251 down-regulated transcripts could be clustered into androgen receptor signalling, formation of the beta-catenin, IL-7 signalling, oestrogen-dependent gene expression, cellular response to retinoic acid, cellular senescence, WNT signalling, regulation of myeloid cell differentiation, nuclear receptor signalling and cytokine activity (Figure 10).

### 3.5. Cholesterol Biosynthesis Is Only Affected by Linoleic Acid and Not by Arachidonic Acid at the Gene Expression Level

**Figure 11:**
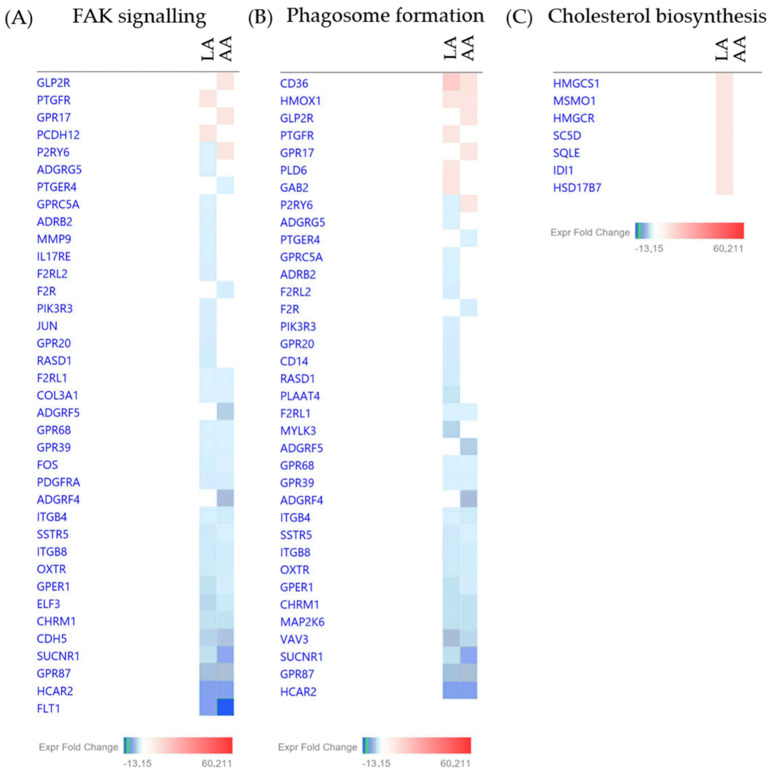
Genes and their expression level changes in LA- or AA-treated SZ95 sebocytes that contributed to the clusters identified by IPA, such as FAK signalling (**A**), phagosome formation (**B**) and cholesterol biosynthesis (**C**). Colour intensities reflect the changes in the expression levels of a given gene in response to either LA or AA treatment.

Using another approach, the ingenuity pathway analysis (IPA), showed that a number of genes related to cholesterol metabolism were induced only by LA, whereas focal adhesion kinase (FAK) signalling and phagosome formation were induced in response to both treatments (Figure 11). 

### 3.6. Linoleic and Arachidonic Acid-Induced Changes in the Lipid Profile of SZ95 Sebocytes 

In order to determine whether, in addition to the changes in the expression levels of genes related to lipid metabolism, there are also differences at the level of lipids, we performed lipidome analyses in SZ95 sebocytes treated with LA and AA for 24 h. In particular, target analysis of FFAs, cholesterol and alpha tocopherol (vitamin E) was performed by GCMS. Neutral lipids, mainly TGs, and polar lipids, mainly PCs, were analysed after separation by reversed-phase and hydrophilic ion-interaction liquid chromatography (LC), respectively. The LC system was coupled to a high-resolution hybrid quadrupole time-of-flight (QTOF) mass spectrometer (MS).

**Figure 12:**
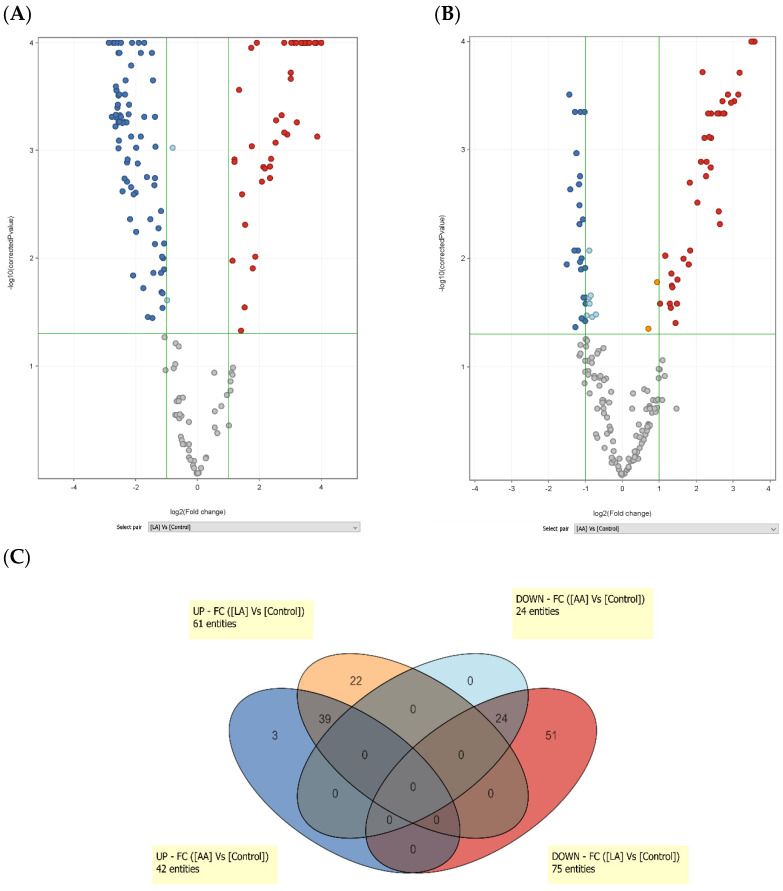
Lipidomic analysis of SZ95 sebocytes treated with LA and AA. Volcano plots showing the changes in lipid abundance in sebocytes treated with LA (**A**) and AA (**B**) compared with vehicle treatment (control). Each dot represents one lipid metabolite. Differential abundance is reported as log2 transformation of fold change (FC) versus control (vehicle). Lipid species that decreased or increased to a statistically different level are indicated by blue and red dots, respectively. Compared with vehicle, LA and AA increases 61 and 42 lipid species, respectively (indicated as entities in the labels). In addition, LA and AA treatments were associated with a decrease of 75 and 24 lipid species (entities), respectively. The identities of the lipids modulated by LA and AA are given in Table S1. (**C**) Venn diagram showing the number of lipid species commonly and specifically modified by LA and AA treatments. The increase of 39 lipid species was shared by LA and AA, while 22 and 3 lipids were specifically increased after treatment with LA and AA, respectively. Fifty-one lipids were uniquely down-regulated by LA, while 24 species were down-regulated by both LA and AA. Lipid identity, modulation, molecular mass, and retention time in the LCMS separation are shown in Table S1.

Lipid metabolites were quantified against iSTD of the same class and the amount obtained in pmol was normalized to the amount of protein. The amounts of the lipids assessed by the three analytical methods applied to each sample were pooled and treated for the interpretation of the results. Firstly, the effects of LA or AA treatments were compared with vehicle (control) using volcano plots (Figure 12). 

The number of individual lipids whose concentration increased or decreased significantly by more than 2-fold after LA treatment was 61 and 75, respectively. Among the increased lipids, TGs were the most numerous (40) followed by diglycerides (DGs, 8) cholesterol esters (CEs, 7), ceramides (4), and free fatty acids (FFAs, 2).

Polar lipids, i.e., PCs (23) and phosphatidylethanolamines (PEs, 19) were the lipid species significantly decreased by LA, followed by sphingomyelins (SMs, 12), FFAs (10), ceramides (6), and one TG (Table S1). AA caused the change in abundance of a smaller number of species. Among the 42 lipids whose concentrations were significantly increased by AA, TGs were the most abundant (32), followed by CEs (7), DGs (2), and one PE. In particular, the AA-containing CE 20:4 and CE 26:5, were abundantly and exclusively increased by AA, suggesting that CEs represent an important reservoir of AA and probably its metabolites. Among the decreased polar species, PCs (11) and PEs (8) were the most affected numerically, followed by SMs (2), one FFA, one ceramide and vitamin E (Table S1). 

Based on the Venn diagram shown in Figure 12C, 39 and 24 lipids were increased and decreased, respectively, by both LA and AA. This observation allowed us to circumscribe the lipid species that were significantly modified in their quantity, independent of the LA or AA source. In Table S1, the lipids are colour coded to distinguish the species that were up-regulated by LA only (orange), AA only (dark pink), and by both LA and AA (light pink). Those that were down-modulated by LA only and by both LA and AA were coloured full and light blue, respectively. An interesting pattern in the distribution of complex lipids, differentiated by the number of double bonds (DBs) and of carbon atoms (C) in TGs was observed. TGs increased by LA only had an average DB and C-number of 3.0 and 51.8, respectively. TGs increased by both LA and AA were characterized by a higher average DB number (5.75), and more C-carbon atoms (average value 54.8). Phospholipids accounted almost exclusively among the lipids that decreased in concentration after LA or AA, which indicates that PCs have a particular role in providing the DG-backbone in the formation of TGs that load the supplied FA in the free form. The evaluation of the molecular characteristics of ceramides (Cer[NS]), whose amounts were increased and decreased after LA supply, also suggested that LA specifically intervened in sphingolipid plasticity. Four Cer[NS] with a total chain length of 32 to 35 C-atoms were increased after LA, whereas the Cer[NS] members with a chain length of 36 to 44 C-atoms were decreased. The Cer[NS] 44:1 was also decreased by AA. 

As shown in detail in Figure S2, the amount of myristic acid (C14:0) showed a robust increase, whereas palmitic acid (C16:0), margaric acid (C17:0), arachidic acid (C20:0), heneicosylic acid (C21:0), and behenic acid (C22:0) were significantly decreased in LA-treated sebocytes compared with AA-treated cells, while the amounts of pentadecylic acid (C15:0) and tricosylic acid (C23:0) decreased similarly. Interestingly, lignoceric acid (C24:0) decreased in LA but increased in the AA-treated sebocytes. Regarding the measured unsaturated lipids, LA significantly decreased the amount of palmitoleic acid (C16:1*n*-7), while the amount of sapienate (C16:1*n*-10) was unchanged. In contrast, AA treatment led to a detectable but not significant decrease in the levels of eicosenoic acid (C20:1) and docosenoic acid (C22:1). 

When cholesterol levels were measured, an increase in cholesterol levels was only detected in the LA-treated cells. These results suggest that, although AA is a metabolite of LA, the two fatty acids have different effects on the lipid production in sebocytes, which may then influence the gene expression profile (Figure S2).

Interestingly, the amounts of FFAs other than C14:0 and C18:2, were greatly reduced after LA treatment, while the net concentration of total FFAs remained unchanged. This observation suggests that exogenous LA is distributed among different classes of intracellular lipids and displaces intracellular FFAs, mainly saturated ones.

In order to understand the interrelationships between the FAs detected in their free form by GCMS and the complex lipids, both neutral and polar, that bind FAs, expression profiles of TGs, ceramides, and phospholipids, mainly including PCs, PEs, and SMs were elaborated in hierarchical clustering. 

As shown in the heat map, the two FAs had a distinct effect on the lipid profile of SZ95 sebocytes, with only a small number of lipids showing similar up- or down-regulation in their levels (Figure S3). The effects on lipid distribution were in the same direction, i.e., decreased levels of polar and neutral lipids, but to a different extent. LA decreased polar lipids to a greater extent than AA (upper part of the heat map). The effects on neutral lipids were more pronounced for TGs and CEs for LA and AA. C18:2 and C14:0 increased consistently and were both positioned next to CE 18:2 and CE 20:0, several TGs, and short-chain ceramides, suggesting that C14:0 is correlated with LA uptake and metabolism in cells. The consistent decrease in vitamin E following both LA and AA could be due to different mechanisms, including conformational changes in the plasma membrane or an increased requirement for lipophilic antioxidants resulting from the uptake of exogenously supplied PUFAs (Table S1). However, the concentration of cholesterol, which is abundant in the plasma membrane, was found to be higher in LA compared with AA-treated cells (Figure S2).

Hierarchical clustering of the lipids measured in SZ95 sebocytes treated with AA or LA for 24 h revealed that the majority of macroscopic changes at the lipid class level after LA and AA treatments were in the same direction, i.e., TGs and CEs increased, while PCs decreased (Figure S3). Notably, individual lipid species were specifically modified depending on the LA or AA lipid supply. In particular LA- and AA-containing CEs, i.e., CE 18:2 and CE 20:4 were direct markers of their respective treatments. On the other hand, enrichment in certain domains of the lipid organization may affect the plasma membrane structure, lipid rafts, and the susceptibility to release signalling molecules [30].

## 4. Discussion

As an essential omega-6 PUFA, LA cannot be synthesized by human cells and is therefore obtained from diet. Sources include nuts, seeds and vegetable oils such as rapeseed, soybean, corn, and sunflower oils, meat and eggs [31,32,33]. In terms of skin homeostasis, as the most abundant PUFA in the epidermis, LA has several beneficial properties such as its maintenance of the integrity of the epidermal water permeability barrier [5,9,34,35]. Indeed, in dry atopic skin, topical application of sunflower seed oil (whose main lipid component is LA) preserved the integrity of the stratum corneum and improved hydration due to increased keratinocyte proliferation, lipid synthesis and efficient activation of PPAR-α [36,37], a key transcription factor responsible for lipid production in sebocytes [21,38]. In the case of acne patients whose sebum and epidermal lipids are characterized by decreased levels of LA [39], a partial explanation may be offered by the finding that LA may be diluted within the increased amount of sebum [14], as sebum was found to diffuse into follicular keratinocytes. It is suggested that the resulting change in the fatty acid component of the normally linoleate-rich epidermal acylceramides, together with follicular hyperkeratinisation and increased transepidermal water loss, contribute to the formation of the acne lesion [14]. 

Based on previous findings, LA may also be a potent inducer of differentiation and lipid metabolism in sebocytes in vitro, increasing lipid droplet size and intracellular levels of neutral lipids by inducing the expression of genes involved in fatty acid metabolism [11,18,40,41]; however, the question remains unanswered as to whether the observed changes are a general response of sebocytes to the increased levels of lipids in the microenvironment or a specific consequence of LA. Overall, just as the available meta-analysis data are controversial regarding its beneficial role/adverse effects in the case of coronary heart disease [42,43,44], and thus the optimal level of LA intake, it is also challenging to answer how central the position of LA is in sebocyte biology. 

In this study we confirmed that LA may have selective effects on SGs already at the level of gene expression regulation. When compared with PA, which along with LA is one of the major FFA in sebum [45], we found that sebocytes are able to differentiate between PA and LA already at the level of gene expression regulation. Only 108 genes involved in basic sebocyte signalling pathways such as MAPK, WNT, leukotriene D4, prostaglandin and retinoic acid signalling, immune cell functions and IL-10 and 17 signalling were regulated by both stimuli [46]. Further investigating the possible regulatory role of LA in SG homeostasis, we found that LA may contribute by regulating genes involved in intracellular fatty acid metabolism, cholesterol biosynthesis, steroid metabolism, regulation of lipid metabolism by PPAR-α, androgen and nuclear receptor signalling and ERK1/2 signalling. These pathways, which are also known to be key regulators of sebocyte functions such as lipid and steroid metabolism, growth and differentiation [21,47,48,49], support the idea that LA is specifically involved in the homeostasis of SGs in terms of differentiation, lipid metabolism and hence sebum production. 

Although an oversimplified conclusion of our results and those of others is that LA exerts numerous beneficial properties in SG biology, it is also the source of AA, the precursor of inflammatory leukotrienes and prostaglandins, which contribute to the inflammatory tissue microenvironment in various diseases such as acne and atopic dermatitis (AD) [21,50]. Therefore, selective modulation of further AA metabolism has already been raised as a therapeutic target to inhibit the enzymatic activities of cyclooxygenases and lipoxygenases such as cyclooxygenase-2 (COX 2) and 5-lipoxygenase (5-LOX), whose expressions are increased in acne skin samples [21]. Indeed, a pilot study has shown that the 5-LOX inhibitor zileuton directly inhibited sebum production by lowering the levels of FFAs and reducing the number of inflammatory skin lesions in acne patients [38]. Therefore, to answer the intriguing question of the role of the LA–AA axis in sebocytes, previous studies have already evaluated the different effects of LA and AA, but our study is the first to compare the changes in the gene expression profiles in LA- and AA-treated SZ95 sebocytes. While, both LA and AA are known to induce the secretion of pro-inflammatory cytokines, e.g., IL-6 and 8 and LA to enhance the innate immune defence in human sebocytes [6,7,17,18], LA inhibited T helper (Th) 1 and Th17 cell differentiation and induced cell death in CD4+ T cells [51,52], promoted the production of thromboxane, TNF-α, COX-2, reactive oxygen species (ROS), IL-8 and matrix metallopeptidase-9 (MMP-9) in neutrophils [53,54,55], and increased the mRNA expression of pro-inflammatory markers (e.g., IL-6, C-C motif chemokine ligand-2 [CCL-2] and IL1-β) in peritoneal macrophages [56]. In contrast with the pro-inflammatory effects, other anti-inflammatory effects have been described, such as the inhibited secretion of IL-1β, IL-6 and TNF-α by LA in *C. acnes*-activated macrophages [5]. In our results, we have shown that a large number of genes showing regulation in response to both LA and AA formed immune-modulatory clusters, e.g., cellular response to TGF-β and TNF, cytokine activity and regulation of the MAPK cascade and leukocyte migration/chemotaxis and differentiation, regulation of IFN-γ and IL-17 signalling, which may be related not only to acne but also to other inflammatory skin diseases [57,58,59]. 

Interestingly, the IL-4/IL-13 signalling pathway, which is a central regulator of many of the hallmark features of AD, and has been found to regulate sex steroid hormone synthesis in human sebocytes and to drive lipid abnormalities in the skin [60], was also affected in SZ95 sebocytes by both LA and AA. Though the cluster forming genes were mostly down-regulated, further studies are needed to answer whether the changes are pro- or anti-inflammatory and how these changes interact with the microenvironment. 

Importantly, while the majority of the immune-related genes were regulated by both LA and AA, our results show that a large number of genes involved in hormone-regulated homeostasis and lipid metabolism were only regulated by LA and not by AA, suggesting that there is an AA-independent effect of LA. One possible explanation is that LA is first converted to γ-linoleic acid and then to dihomo-γ-linoleic acid, the intermediates of which may also have regulatory effects on sebocyte gene expression [61]. Interestingly, histopathological findings have shown that administration of γ-linoleic acid to acne patients reduced follicular hyperkeratinisation and inflammation and improved acne lesions through modulation of the protein kinase C (PKC)/MAPK pathway [62]. 

Bearing in mind the limitations of linking gene expression data to protein levels and especially enzyme activity, our mRNA data still strongly suggest that key lipid metabolism-related pathways are also selectively regulated by LA and AA. Therefore, to assess whether changes and differences might also be induced at the lipid level, we examined the lipid profiles in LA- and AA-treated sebocytes, which have only been investigated to a limited extent in previous studies. The differences observed—in particular that LA but not AA treatment significantly decreased the levels of selected saturated fatty acids while increasing the levels of cholesterol—suggest that the lipid signature is also dependent on the fatty acid source. Interestingly, there were distinct effects on storage lipids (TGs), plasma membrane lipids (PCs, PEs), lipid rafts and signalling lipids (cholesterol esters, CEs and ceramides). These findings may therefore complement previous observations in which LA caused down-modulation of the epidermal growth factor receptor (EGFR and ERBBs) [63]. Down-regulation of these pathways by siRNAs in SZ95 sebocytes resulted in increased levels of TGs, and CEs, particularly CE 18:2 and CE 20:4 [63].

The storage of neutral lipids is related to the shaping of lipids in the plasma membrane. Interestingly, LA decreased polar lipids to a greater extent than AA. While this could support an increase in free C14:0 as a result of displacement by LA, we found no correlation between C14:0 and changes in polar lipids. Thus, other mechanisms are involved in the elevation of free C14:0. 

Given the wide range of their biological functions, the role of ceramides in sebocytes is underexplored, although impairment of ceramide synthesis has been observed in parallel with sebaceous lipid disruption when perilipin 2 (PLIN2), a key lipid droplet associated protein, is silenced in SZ95 sebocytes [10]. Our observations suggest that LA may have a greater capacity to initiate ceramide de novo synthesis, as evidenced by the increased levels of short-chain ceramide members. These findings call for further studies to answer whether such an effect is due to LA itself or to the associated increase in C14:0 levels. Indeed, ceramide de novo synthesis is initiated by FAs, typically palmitate, which is conjugated with serine by serine-palmitoyl-transferase (SPT) to form C18-sphingosine, but C14:0 can also be used by SPT to form C16-sphingosine, which is then conjugated with FAs to form ceramides with 2-carbon shorter sphingoid bases [10,64]. 

## 5. Conclusions

In conclusion, these results strongly support the idea that LA is a key regulator of sebocyte biology, influencing immune processes, differentiation and lipid production at the level of gene expression. In addition, we have shown that LA may have a complex effect both as a precursor of AA and independently by modifying the levels of other biologically active lipids. Our findings could therefore be applied to the development and selection of therapies that modulate lipid production and selectively target AA metabolism in sebocytes.

## Data Availability

RNA-Seq data are available in the Sequence Read Archive (SRA) database under the following accession numbers: PRJNA646337 for PA and PRJNA882620 for LA and AA.

## Supplementary Materials

The following supporting information can be downloaded at: https://www.mdpi.com/article/10.3390/nu15153315/s1, Figure S1: Heat map showing the replicates separately in LA and AA-treated SZ95 sebocytes for 24 h as determined by our RNA-seq analysis. Figure S2: Determination of selected saturated and unsaturated fatty acids and cholesterol levels by GCMS in SZ95 sebocytes treated with LA or AA for 24 h; Figure S3: Hierarchical clustering tree of the measured lipids in SZ95 sebocytes treated with AA or LA for 24 h; Table S1: Lipid metabolites significantly altered by LA and AA treatments. The list includes lipid identity, modulation, molecular mass, and retention time in the LCMS system.

## References

[1] Zouboulis C.C., Baron J.M., Böhm M., Kippenberger S., Kurzen H., Reichrath J., Thielitz A. (2008). Frontiers in sebaceous gland biology and pathology. Exp. Dermatol..

[2] Fluhr J.W., Mao-Qiang M., Brown B.E., Wertz P.W., Crumrine D., Sundberg J.P., Feingold K.R., Elias P.M. (2003). Glycerol regulates stratum corneum hydration in sebaceous gland deficient (asebia) mice. J. Investig. Dermatol..

[3] Picardo M., Zompetta C., De Luca C., Cirone M., Faggioni A., Nazzaro-Porro M., Passi S., Prota G. (1991). Role of skin surface lipids in UV-induced epidermal cell changes. Arch. Dermatol. Res..

[4] Lovászi M., Szegedi A., Zouboulis C.C., Törőcsik D. (2017). Sebaceous-immunobiology is orchestrated by sebum lipids. Derm. Endocrinol..

[5] Lovaszi M., Mattii M., Eyerich K., Gacsi A., Csanyi E., Kovacs D., Ruhl R., Szegedi A., Kemeny L., Stahle M. (2017). Sebum lipids influence macrophage polarization and activation. Br. J. Dermatol..

[6] Törőcsik D., Fazekas F., Póliska S., Gregus A., Janka E.A., Dull K., Szegedi A., Zouboulis C.C., Kovács D. (2021). Epidermal Growth Factor Modulates Palmitic Acid-Induced Inflammatory and Lipid Signaling Pathways in SZ95 Sebocytes. Front. Immunol..

[7] Nakatsuji T., Kao M.C., Zhang L., Zouboulis C.C., Gallo R.L., Huang C.M. (2010). Sebum free fatty acids enhance the innate immune defense of human sebocytes by upregulating beta-defensin-2 expression. J. Investig. Dermatol..

[8] Wille J.J., Kydonieus A. (2003). Palmitoleic acid isomer (C16:1delta6) in human skin sebum is effective against gram-positive bacteria. Ski. Pharmacol. Appl. Ski. Physiol..

[9] Elias P.M., Brown B.E., Ziboh V.A. (1980). The permeability barrier in essential fatty acid deficiency: Evidence for a direct role for linoleic acid in barrier function. J. Investig. Dermatol..

[10] Dahlhoff M., Camera E., Picardo M., Zouboulis C.C., Chan L., Chang B.H., Schneider M.R. (2013). PLIN2, the major perilipin regulated during sebocyte differentiation, controls sebaceous lipid accumulation in vitro and sebaceous gland size in vivo. Biochim. Biophys. Acta.

[11] Chen W., Yang C.C., Sheu H.M., Seltmann H., Zouboulis C.C. (2003). Expression of peroxisome proliferator-activated receptor and CCAAT/enhancer binding protein transcription factors in cultured human sebocytes. J. Investig. Dermatol..

[12] Zouboulis C.C., Hossini A.M., Hou X., Wang C., Weylandt K.H., Pietzner A. (2023). Effects of Moringa oleifera Seed Oil on Cultured Human Sebocytes In Vitro and Comparison with Other Oil Types. Int. J. Mol. Sci..

[13] Ottaviani M., Camera E., Picardo M. (2010). Lipid mediators in acne. Mediat. Inflamm..

[14] Downing D.T., Stewart M.E., Wertz P.W., Strauss J.S. (1986). Essential fatty acids and acne. J. Am. Acad. Dermatol..

[15] Letawe C., Boone M., Piérard G.E. (1998). Digital image analysis of the effect of topically applied linoleic acid on acne microcomedones. Clin. Exp. Dermatol..

[16] Valacchi G., De Luca C., Wertz P.W. (2010). Lipid mediators in skin inflammation: Updates and current views. Mediat. Inflamm..

[17] Choi C.W., Kim Y., Kim J.E., Seo E.Y., Zouboulis C.C., Kang J.S., Youn S.W., Chung J.H. (2019). Enhancement of lipid content and inflammatory cytokine secretion in SZ95 sebocytes by palmitic acid suggests a potential link between free fatty acids and acne aggravation. Exp. Dermatol..

[18] Zouboulis C.C., Angres S., Seltmann H. (2011). Regulation of stearoyl-coenzyme A desaturase and fatty acid delta-6 desaturase-2 expression by linoleic acid and arachidonic acid in human sebocytes leads to enhancement of proinflammatory activity but does not affect lipogenesis. Br. J. Dermatol..

[19] Dozsa A., Dezso B., Toth B.I., Bacsi A., Poliska S., Camera E., Picardo M., Zouboulis C.C., Bíró T., Schmitz G. (2014). PPARγ-mediated and arachidonic acid-dependent signaling is involved in differentiation and lipid production of human sebocytes. J. Investig. Dermatol..

[20] Zouboulis C.C., Coenye T., He L., Kabashima K., Kobayashi T., Niemann C., Nomura T., Oláh A., Picardo M., Quist S.R. (2022). Sebaceous immunobiology—Skin homeostasis, pathophysiology, coordination of innate immunity and inflammatory response and disease associations. Front. Immunol..

[21] Alestas T., Ganceviciene R., Fimmel S., Muller-Decker K., Zouboulis C.C. (2006). Enzymes involved in the biosynthesis of leukotriene B4 and prostaglandin E2 are active in sebaceous glands. J. Mol. Med..

[22] Oh S.Y., Lee S.J., Jung Y.H., Lee H.J., Han H.J. (2015). Arachidonic acid promotes skin wound healing through induction of human MSC migration by MT3-MMP-mediated fibronectin degradation. Cell Death Dis..

[23] Ge L., Gordon J.S., Hsuan C., Stenn K., Prouty S.M. (2003). Identification of the delta-6 desaturase of human sebaceous glands: Expression and enzyme activity. J. Investig. Dermatol..

[24] Zouboulis C.C., Seltmann H., Neitzel H., Orfanos C.E. (1999). Establishment and characterization of an immortalized human sebaceous gland cell line (SZ95). J. Investig. Dermatol..

[25] Bindea G., Mlecnik B., Hackl H., Charoentong P., Tosolini M., Kirilovsky A., Fridman W.H., Pages F., Trajanoski Z., Galon J. (2009). ClueGO: A Cytoscape plug-in to decipher functionally grouped gene ontology and pathway annotation networks. Bioinformatics.

[26] Törőcsik D., Kovács D., Camera E., Lovászi M., Cseri K., Nagy G.G., Molinaro R., Rühl R., Tax G., Szabó K. (2014). Leptin promotes a proinflammatory lipid profile and induces inflammatory pathways in human SZ95 sebocytes. Br. J. Dermatol..

[27] Ludovici M., Kozul N., Materazzi S., Risoluti R., Picardo M., Camera E. (2018). Influence of the sebaceous gland density on the stratum corneum lipidome. Sci. Rep..

[28] Isseroff R.R., Ziboh V.A., Chapkin R.S., Martinez D.T. (1987). Conversion of linoleic acid into arachidonic acid by cultured murine and human keratinocytes. J. Lipid Res..

[29] Chapkin R.S., Ziboh V.A., Marcelo C.L., Voorhees J.J. (1986). Metabolism of essential fatty acids by human epidermal enzyme preparations: Evidence of chain elongation. J. Lipid Res..

[30] Mathay C., Pierre M., Pittelkow M.R., Depiereux E., Nikkels A.F., Colige A., Poumay Y. (2011). Transcriptional profiling after lipid raft disruption in keratinocytes identifies critical mediators of atopic dermatitis pathways. J. Investig. Dermatol..

[31] Saini R.K., Keum Y.S. (2018). Omega-3 and omega-6 polyunsaturated fatty acids: Dietary sources, metabolism, and significance—A review. Life Sci..

[32] Innes J.K., Calder P.C. (2018). Omega-6 fatty acids and inflammation. Prostaglandins Leukot. Essent. Fat. Acids.

[33] Whelan J., Fritsche K. (2013). Linoleic acid. Adv. Nutr..

[34] Cho Y., Ziboh V.A. (1994). Incorporation of 13-hydroxyoctadecadienoic acid (13-HODE) into epidermal ceramides and phospholipids: Phospholipase C-catalyzed release of novel 13-HODE-containing diacylglycerol. J. Lipid Res..

[35] Hansen H.S., Jensen B. (1985). Essential function of linoleic acid esterified in acylglucosylceramide and acylceramide in maintaining the epidermal water permeability barrier. Evidence from feeding studies with oleate, linoleate, arachidonate, columbinate and alpha-linolenate. Biochim. Biophys. Acta.

[36] Danby S.G., AlEnezi T., Sultan A., Lavender T., Chittock J., Brown K., Cork M.J. (2013). Effect of olive and sunflower seed oil on the adult skin barrier: Implications for neonatal skin care. Pediatr. Dermatol..

[37] Hanley K., Jiang Y., He S.S., Friedman M., Elias P.M., Bikle D.D., Williams M.L., Feingold K.R. (1998). Keratinocyte differentiation is stimulated by activators of the nuclear hormone receptor PPARalpha. J. Investig. Dermatol..

[38] Zouboulis Ch C., Saborowski A., Boschnakow A. (2005). Zileuton, an oral 5-lipoxygenase inhibitor, directly reduces sebum production. Dermatology.

[39] Pappas A., Johnsen S., Liu J.C., Eisinger M. (2009). Sebum analysis of individuals with and without acne. Derm. Endocrinol..

[40] Hong I., Lee M.H., Na T.Y., Zouboulis C.C., Lee M.O. (2008). LXRalpha enhances lipid synthesis in SZ95 sebocytes. J. Investig. Dermatol..

[41] Makrantonaki E., Zouboulis C.C. (2007). Testosterone metabolism to 5alpha-dihydrotestosterone and synthesis of sebaceous lipids is regulated by the peroxisome proliferator-activated receptor ligand linoleic acid in human sebocytes. Br. J. Dermatol..

[42] Farvid M.S., Ding M., Pan A., Sun Q., Chiuve S.E., Steffen L.M., Willett W.C., Hu F.B. (2014). Dietary linoleic acid and risk of coronary heart disease: A systematic review and meta-analysis of prospective cohort studies. Circulation.

[43] Chowdhury R., Warnakula S., Kunutsor S., Crowe F., Ward H.A., Johnson L., Franco O.H., Butterworth A.S., Forouhi N.G., Thompson S.G. (2014). Association of dietary, circulating, and supplement fatty acids with coronary risk: A systematic review and meta-analysis. Ann. Intern. Med..

[44] Ramsden C.E., Zamora D., Majchrzak-Hong S., Faurot K.R., Broste S.K., Frantz R.P., Davis J.M., Ringel A., Suchindran C.M., Hibbeln J.R. (2016). Re-evaluation of the traditional diet-heart hypothesis: Analysis of recovered data from Minnesota Coronary Experiment (1968–1973). BMJ Clin. Res. Ed..

[45] Pappas A., Anthonavage M., Gordon J.S. (2002). Metabolic fate and selective utilization of major fatty acids in human sebaceous gland. J. Investig. Dermatol..

[46] Zouboulis C.C., Picardo M., Ju Q., Kurokawa I., Torocsik D., Biro T., Schneider M.R. (2016). Beyond acne: Current aspects of sebaceous gland biology and function. Rev. Endocr. Metab. Disord..

[47] Fritsch M., Orfanos C.E., Zouboulis C.C. (2001). Sebocytes are the key regulators of androgen homeostasis in human skin. J. Investig. Dermatol..

[48] Zouboulis C.C. (2004). Acne and sebaceous gland function. Clin. Dermatol..

[49] Schneider M.R., Paus R. (2010). Sebocytes, multifaceted epithelial cells: Lipid production and holocrine secretion. Int. J. Biochem. Cell Biol..

[50] Yanes D.A., Mosser-Goldfarb J.L. (2018). Emerging therapies for atopic dermatitis: The prostaglandin/leukotriene pathway. J. Am. Acad. Dermatol..

[51] Huang X., Yi S., Hu J., Du Z., Wang Q., Ye Z., Su G., Kijlstra A., Yang P. (2021). Linoleic acid inhibits in vitro function of human and murine dendritic cells, CD4(+)T cells and retinal pigment epithelial cells. Graefe’s Arch. Clin. Exp. Ophthalmol..

[52] Ma C., Kesarwala A.H., Eggert T., Medina-Echeverz J., Kleiner D.E., Jin P., Stroncek D.F., Terabe M., Kapoor V., ElGindi M. (2016). NAFLD causes selective CD4(+) T lymphocyte loss and promotes hepatocarcinogenesis. Nature.

[53] Vaughan J.E., Walsh S.W. (2005). Neutrophils from pregnant women produce thromboxane and tumor necrosis factor-alpha in response to linoleic acid and oxidative stress. Am. J. Obstet. Gynecol..

[54] Hatanaka E., Levada-Pires A.C., Pithon-Curi T.C., Curi R. (2006). Systematic study on ROS production induced by oleic, linoleic, and gamma-linolenic acids in human and rat neutrophils. Free. Radic. Biol. Med..

[55] Mena S.J., Manosalva C., Carretta M.D., Teuber S., Olmo I., Burgos R.A., Hidalgo M.A. (2016). Differential free fatty acid receptor-1 (FFAR1/GPR40) signalling is associated with gene expression or gelatinase granule release in bovine neutrophils. Innate Immun..

[56] Kain V., Halade G.V. (2019). Immune responsive resolvin D1 programs peritoneal macrophages and cardiac fibroblast phenotypes in diversified metabolic microenvironment. J. Cell. Physiol..

[57] Navarini A.A., Simpson M.A., Weale M., Knight J., Carlavan I., Reiniche P., Burden D.A., Layton A., Bataille V., Allen M. (2014). Genome-wide association study identifies three novel susceptibility loci for severe *Acne vulgaris*. Nat. Commun..

[58] Kelhala H.L., Palatsi R., Fyhrquist N., Lehtimaki S., Vayrynen J.P., Kallioinen M., Kubin M.E., Greco D., Tasanen K., Alenius H. (2014). IL-17/Th17 pathway is activated in acne lesions. PLoS ONE.

[59] Mattii M., Lovászi M., Garzorz N., Atenhan A., Quaranta M., Lauffer F., Konstantinow A., Küpper M., Zouboulis C.C., Kemeny L. (2018). Sebocytes contribute to skin inflammation by promoting the differentiation of T helper 17 cells. Br. J. Dermatol..

[60] Zhang C., Chinnappan M., Prestwood C.A., Edwards M., Artami M., Thompson B.M., Eckert K.M., Vale G., Zouboulis C., McDonald J.G. (2021). Interleukins 4 and 13 drive lipid abnormalities in skin cells through regulation of sex steroid hormone synthesis. Proc. Natl. Acad. Sci. USA.

[61] Balić A., Vlašić D., Žužul K., Marinović B., Bukvić Mokos Z. (2020). Omega-3 Versus Omega-6 Polyunsaturated Fatty Acids in the Prevention and Treatment of Inflammatory Skin Diseases. Int. J. Mol. Sci..

[62] Jung J.Y., Kwon H.H., Hong J.S., Yoon J.Y., Park M.S., Jang M.Y., Suh D.H. (2014). Effect of dietary supplementation with omega-3 fatty acid and gamma-linolenic acid on acne vulgaris: A randomised, double-blind, controlled trial. Acta Derm. Venereol..

[63] Dahlhoff M., Camera E., Ludovici M., Picardo M., Muller U., Leonhardt H., Zouboulis C.C., Schneider M.R. (2015). EGFR/ERBB receptors differentially modulate sebaceous lipogenesis. FEBS Lett..

[64] Holleran W.M., Williams M.L., Gao W.N., Elias P.M. (1990). Serine-palmitoyl transferase activity in cultured human keratinocytes. J. Lipid Res..

