# Supplementary material for: Linoleic Acid Induced Changes in SZ95 Sebocytes—Comparison with Palmitic Acid and Arachidonic Acid
Source: Nutrients. 2023 Jul 26;15(15):3315. doi: 10.3390/nu15153315 (PMC10420848; doi:10.3390/nu15153315)
Supplement: Supplementary file 1 [file nutrients-15-03315-s001.zip › Supplementary Figure S1.pdf]

Supplementary Figure S1.

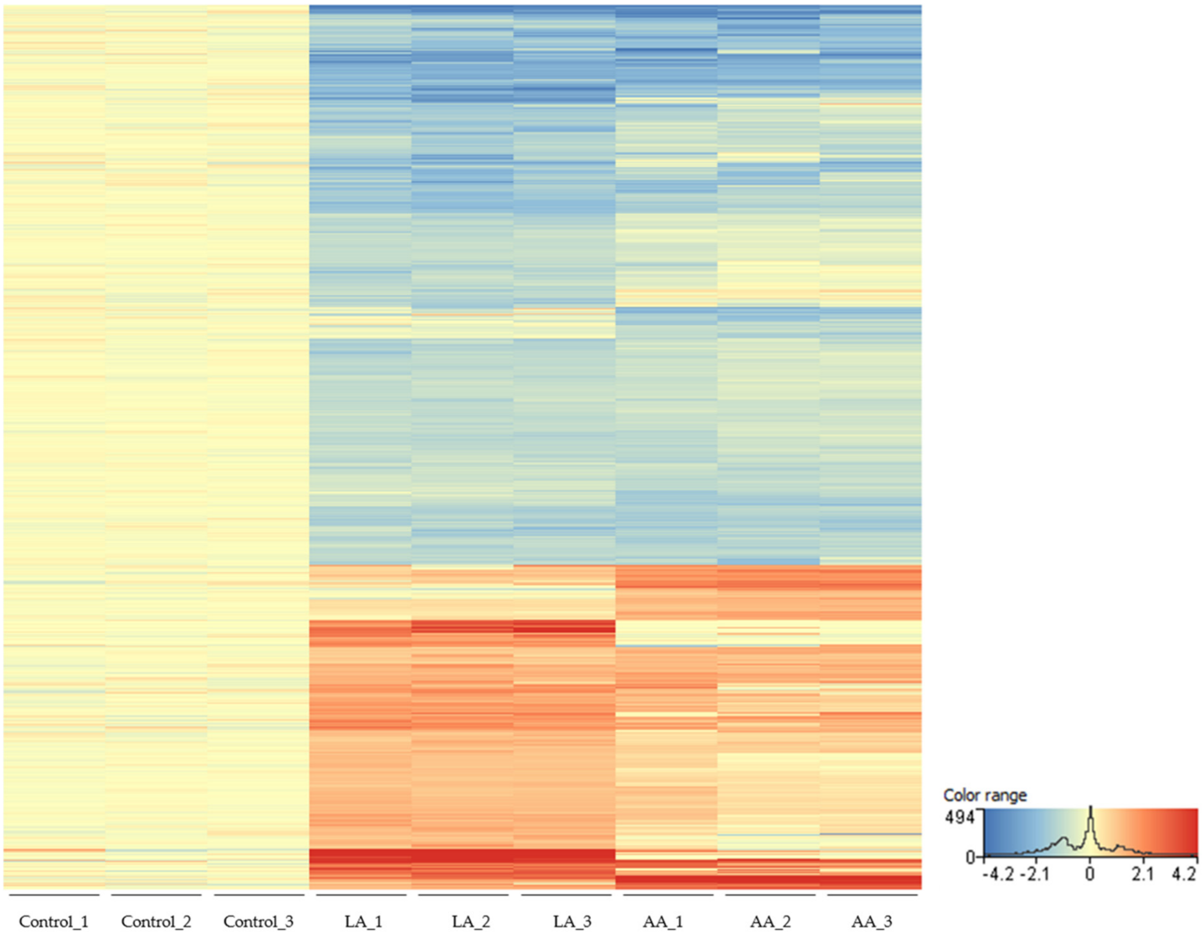

Supplementary Figure S1. Heat map showing the replicates separately to support the heat map in Figure 5, which shows mean averages. Color intensities reflect the ratio of signal intensities as shown.
