# Supplementary material for: Linoleic Acid Induced Changes in SZ95 Sebocytes—Comparison with Palmitic Acid and Arachidonic Acid
Source: Nutrients. 2023 Jul 26;15(15):3315. doi: 10.3390/nu15153315 (PMC10420848; doi:10.3390/nu15153315)
Supplement: Supplementary file 1 [file nutrients-15-03315-s001.zip › Supplementary Figure S2.pdf]

Supplementary Figure S2.

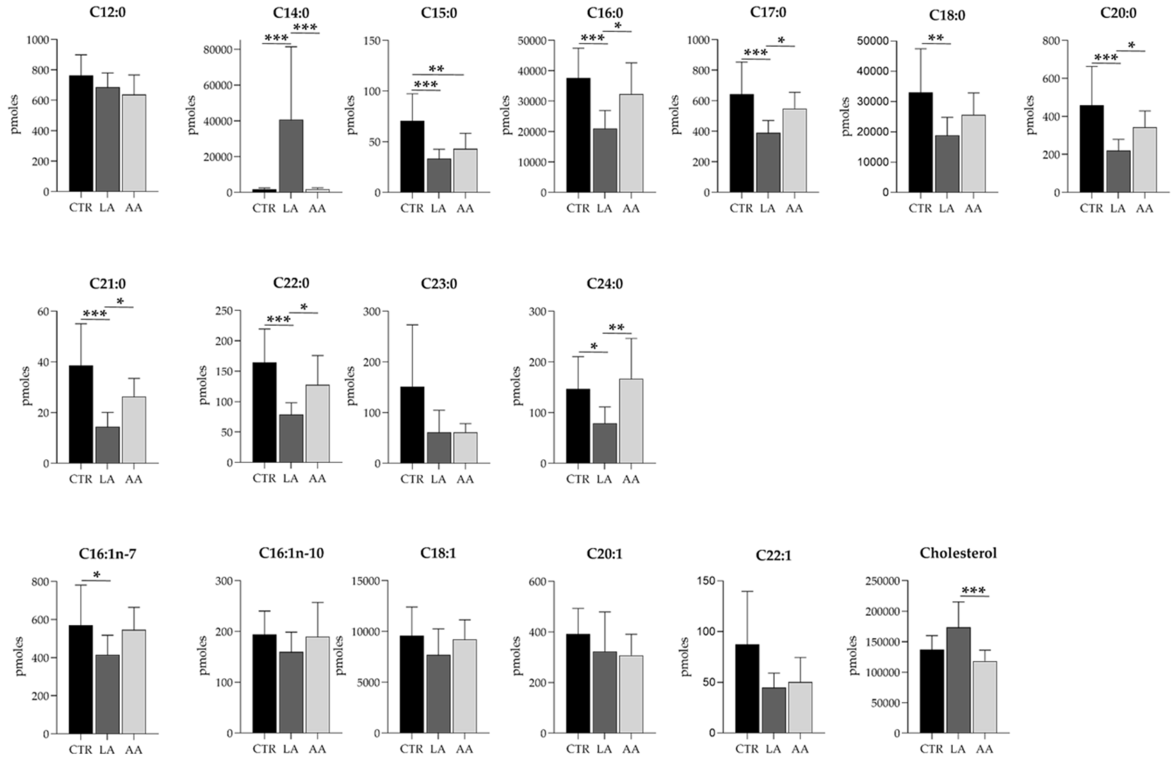

Supplementary Figure S2. Determination of selected saturated and unsaturated fatty acids and cholesterol levels by GCMS in SZ95 sebocytes treated with LA or AA for 24 h. One-way ANOVA followed by Tukey's post hoc test and Kruskal-Wallis test followed by Dunn's post hoc test were used for lipid analysis. Numbers reported are pmol normalised to the OD values of the Bradford assay for proteins performed on cell lysates. * $p \leq 0.05$; ** $p \leq 0.01$; *** $p \leq 0.001$
