# Supplementary material for: Linoleic Acid Induced Changes in SZ95 Sebocytes—Comparison with Palmitic Acid and Arachidonic Acid
Source: Nutrients. 2023 Jul 26;15(15):3315. doi: 10.3390/nu15153315 (PMC10420848; doi:10.3390/nu15153315)
Supplement: Supplementary file 1 [file nutrients-15-03315-s001.zip › Supplementary Figure S3.pdf]

Supplementary Figure S3.

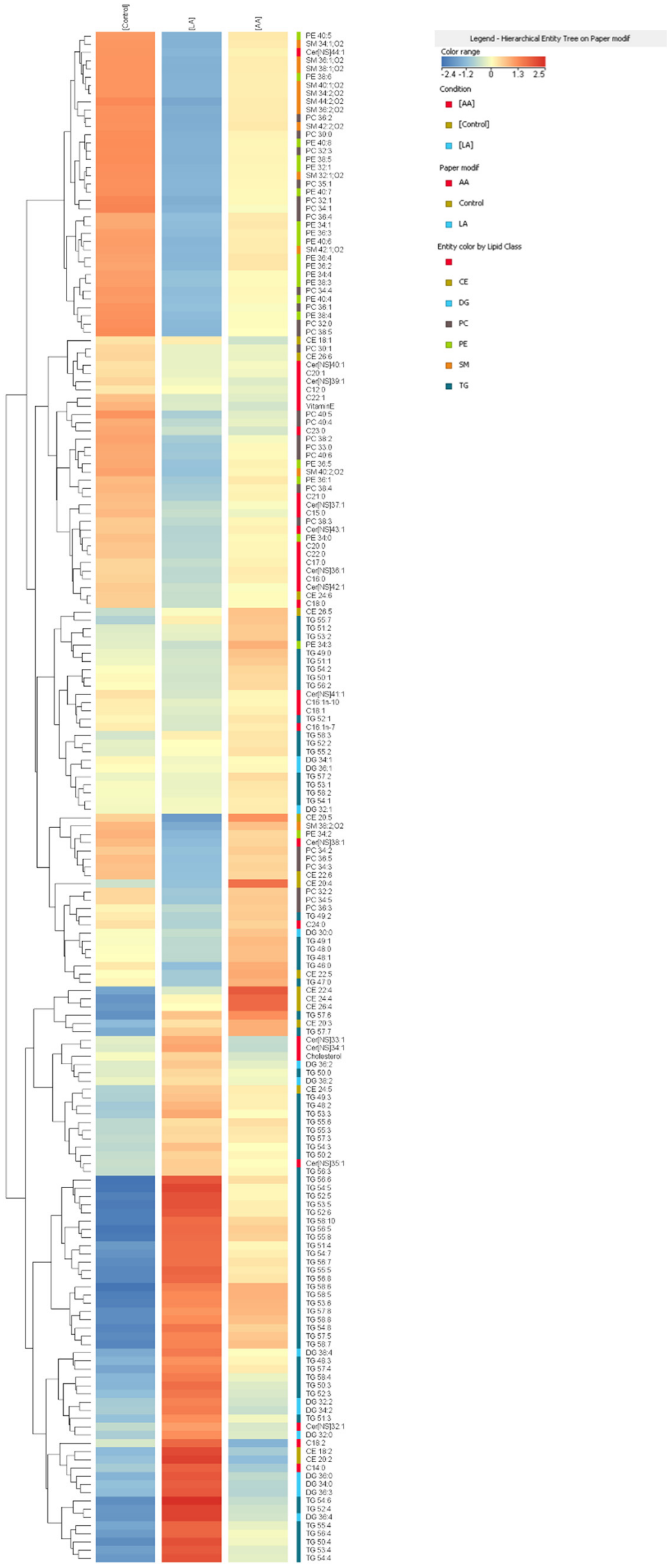

Supplementary Figure S3. Hierarchical clustering tree of measured lipids in SZ95 sebocytes treated with AA or LA for 24 h. Note that the majority of lipids changed their amounts in opposite

directions, while the lipids that were up- or down-regulated in both treatment conditions are enlarged in brackets.

Clustering:
Experiment: SZ95_All Lipidomics Data
Clustering Algorithm: Hierarchical
Clustered by: Normalized intensity values
Clustered On: Entities
Similarity Measure: Euclidean
Linkage Rule: Wards
